# Atorvastatin as an immunomodulatory adjunct in ulcerative colitis, beyond lipid lowering to inflammation control: a randomized controlled pilot study

**DOI:** 10.3389/fphar.2025.1690513

**Published:** 2025-10-29

**Authors:** Mohannad O. Khrieba, Furqan M. Abdulelah, Nada A. Alsaleh, Hayam Ali AlRasheed, Tarek I. Ahmed, Azza El-Sayed Mansy, Manal A. Hamouda, Eslam Habba, Nora Elshorbagi, Ahmed G. Abd Elhameed, Eman Hamza, Muhammed M. Salahuddin, Shereen A. Mourad, Marwa Kamal

**Affiliations:** ^1^ Pharmacy Practice Department, Faculty of Pharmacy, Horus University-Egypt, Damietta, Egypt; ^2^ Department of Clinical Pharmacy, College of Pharmacy, Al-Naji University, Baghdad, Iraq; ^3^ Department of Pharmacy Practice, College of Pharmacy, Princess Nourah bint Abdulrahman University, Riyadh, Saudi Arabia; ^4^ Internal Medicine Department, Faculty of Medicine, Fayoum University, Fayoum, Egypt; ^5^ Clinical Pharmacy Department, Faculty of Pharmacy, Menofia University, Shebeen Elkoum, Egypt; ^6^ Tropical Medicine and Infectious Diseases Department, Faculty of Medicine, Tanta University, Tanta, Egypt; ^7^ Pharmacology and Toxicology Department, Faculty of Pharmacy, Sinai University, Alqantara Branch, Ismailia, Egypt; ^8^ Pharmacology and Toxicology Department, Faculty of Pharmacy, Mansoura University, Mansoura, Egypt; ^9^ Pharmacology and Biochemistry Department, Faculty of Pharmacy, Horus University-Egypt, Damietta, Egypt; ^10^ Department of Biochemistry, College of Medicine, Imam Mohammad Ibn Saud Islamic University (IMSIU), Riyadh, Saudi Arabia; ^11^ Clinical Pathology Department, Faculty of Medicine, Mansoura University, Mansoura, Egypt; ^12^ Clinical Pharmacy Department, Faculty of Pharmacy, Fayoum University, Fayoum, Egypt

**Keywords:** atorvastatin, crp, esr, IBDQ-32, SCCAI, ulcerative colitis

## Abstract

**Background:**

Ulcerative colitis (UC) is a long-term condition marked by recurrent episodes of inflammation affecting the colonic mucosa. Despite mesalamine’s or 5-amino salicylic acid’s (5-ASA) established role in inducing and maintaining remission, some patients experience persistent symptoms and inflammatory activity. Atorvastatin has pleiotropic anti-inflammatory effects, and provide therapeutic benefits in UC. Several preclinical studies assessed the beneficial role of atorvastatin in colitis, but clinical data remain scarce.

**Aim:**

To evaluate the efficacy and safety of 5-ASA plus atorvastatin (versus 5-ASA plus placebo) in patients with mild to moderate UC.

**Methods:**

In this randomized, double-blind Pilot trial, 54 patients with mild-to-moderate UC were randomized to receive 5-ASA plus atorvastatin (Atorvastatin group, n = 27) or 5-ASA plus placebo (Control group, n = 27) for 6 months. Clinical activity was assessed using the Simple Clinical Colitis Activity Index (SCCAI), quality of life using the Inflammatory Bowel Disease Questionnaire (IBDQ-32), and inflammatory status using serum interleukin-18 (IL-18), C-reactive protein (CRP), and erythrocyte sedimentation rate (ESR). Statistical analysis was conducted using intention to treat.

**Results:**

After treatment, both groups showed significant changes in all measured parameters when compared to baseline except for bowel frequency at night in control group (p = 0.148). When compared to the control group, the atorvastatin group demonstrated significantly greater post-treatment improvements in IBDQ systemic (p = 0.001), digestive (p = 0.013), emotional domains (p = 0.015), and total score (p = 0.003). Reductions in IL-18, CRP, and ESR were observed in both groups, but were significantly greater with atorvastatin (IL-18: p = 0.026; CRP: p = 0.027; ESR: p = 0.03, SCCAI: p = 0.0005). Clinical response was achieved in 66.6% of atorvastatin-treated patients versus 44% of controls (p = 0.02). Spearman’s analysis showed IBDQ-32 scores were negatively correlated with SCCAI (r = −0.498), ESR (r = −0.549), CRP (r = −0.356), and IL-18 (r = −0.548). No significant reported side effects.

**Conclusion:**

Adjunctive atorvastatin with 5-ASA significantly improved clinical disease activity, quality of life, and inflammatory biomarkers compared to 5-ASA alone in mild-to-moderate patients with UC.

**Clinical Trial Registration:**

clinicaltrials.gov, Identifier NCT05567068.

## 1 Introduction

Ulcerative colitis (UC) is a persistent, idiopathic form of inflammatory bowel disease (IBD) marked by uninterrupted inflammation of the colonic mucosa, beginning in the rectum and progressing proximally to different lengths of the colon (AlRasheed et al., 2025a). Clinically, it manifests with diarrhea, rectal bleeding, urgency, abdominal pain, and systemic symptoms, which impair daily functioning and health-related quality of life (Alherz et al., 2025). UC follows a relapsing–remitting course, and although medical advances have improved outcomes, many patients still face recurrent disease exacerbations and develop chronic complications. Worldwide, both the incidence and prevalence of UC have shown a steady rise in recent decades, particularly in industrializing nations, suggesting an interplay of genetic predisposition, environmental triggers, and lifestyle changes in its pathogenesis (Bahaa et al., 2024).

Ulcerative colitis pathogenesis is driven by immune system dysregulation triggered by luminal antigens in individuals predisposed by genetic factors (El-Haggar et al., 2024). Barrier dysfunction in the colonic epithelium allows increased antigen penetration, stimulating aberrant activation of innate and adaptive immune pathways. This leads to the overproduction of pro-inflammatory mediators such as interleukin (IL)-1β, tumor necrosis factor-α (TNF-α), IL-6, and IL-18. IL-18, in particular, has a major role in amplifying mucosal inflammation through activation of Th1 and Th17 pathways. Persistent cytokine production perpetuates inflammation, impairs mucosal healing, and drives disease chronicity (Binsaleh et al., 2025).

Atorvastatin, an inhibitor of 3-hydroxy-3-methylglutaryl coenzyme A (HMG-CoA) reductase, is commonly prescribed for managing hyperlipidemia and lowering cardiovascular risk (Boyarintsev et al., 2024). In addition to its lipid-lowering action, it has diverse effects such as enhancing endothelial function, decreasing oxidative stress, and regulating inflammatory processes (Liu et al., 2024). Multiple animal studies have proven that statins exert anti-inflammatory and mucosal-protective effects in experimental models of colitis (Lee et al., 2007; Sasaki et al., 2003b; Soliman et al., 2019). In experimental models of colitis induced by dextran sulfate sodium (DSS) and trinitrobenzene sulfonic acid (TNBS), statin treatment markedly lowered disease activity index values, minimized colonic ulcer formation, and alleviated histological signs of inflammation (Maheshwari et al., 2015; Rashidian et al., 2016; Saber et al., 2021). Mechanistically, these effects are mediated through inhibition of NF-κB activation, suppression of inflammatory markers (including TNF-α, IL-1β, IL-6, and IL-18), reduction of oxidative stress, and improvement of endothelial function (Mansouri et al., 2022; Rashidian et al. 2016; Zivkovic et al., 2023). Statins have also been shown to enhance epithelial barrier integrity by upregulating tight junction proteins and to modulate immune cell infiltration in colonic tissue (El-Mahdy et al., 2021; Vital et al., 2025). Atorvastatin, simvastatin, and pravastatin have all shown beneficial effects in rodent models, with atorvastatin in particular demonstrating a dose-dependent reduction in colonic cytokine levels and myeloperoxidase activity (Alarfaj et al., 2025; Lee et al. 2007; Maheshwari et al. 2015). Observational clinical studies suggest a potential association between statin use and reduced disease activity or hospitalization risk in IBD (Crockett et al., 2012; Khalili et al., 2025; Ungaro et al., 2016), but randomized controlled trial data remain limited (AlRasheed et al., 2025b). These findings support the hypothesis that statins, beyond their lipid-lowering action, may modulate key inflammatory pathways involved in colitis pathogenesis (Vital et al., 2023). Some clinical studies were conducted to evaluate the adjunctive role of atorvastatin in UC, but they did not assess the effect of atorvastatin on quality of life, inflammatory bowel disease questionnaire (IBDQ-32), and simple colitis clinical activity index (SCCAI).

Based on this background, we hypothesized that addition of atorvastatin to 5-ASA therapy in mild-to-moderate UC will result in greater improvements in clinical activity scores, disease-specific quality of life, and inflammatory biomarkers compared to 5-ASA alone, without significant safety concerns. The aim of this study was to evaluate the efficacy and safety of adding atorvastatin to standard 5-ASA therapy in patients with active UC, using validated clinical indices, disease-specific quality of life measures, and laboratory inflammatory markers.

## 2 Patients and methods

From August 2023 to June 2025, fifty-four eligible participants were recruited from the Internal Medicine Department, Faculty of Medicine, Fayoum University. The Institutional Review Board approved the study (Approval No. M 417), and all procedures adhered to the principles of the Helsinki Declaration. Participation was voluntary, with the option to withdraw at any stage. Both investigators and patients remained blinded to the assigned treatments.

### 2.1 Inclusion criteria

Eligible patients were adults (≥18 years) of either gender with an endoscopically confirmed diagnosis of mild to moderate UC. Both 5-ASA-naïve patients and those already receiving 5-ASA therapy were included.

### 2.2 Exclusion criteria

The study excluded patients with severe forms of ulcerative colitis, as well as those who had been treated with systemic or topical corticosteroids or any immunosuppressive agents. Individuals with hepatic or renal impairment were omitted to avoid potential metabolic complications related to atorvastatin therapy. Participants with a prior diagnosis of hyperlipidemia, musculoskeletal disorders, colorectal cancer, or those who had undergone partial or total colectomy were also not considered eligible. In addition, pregnant women and any participants with known hypersensitivity to the investigational drugs were excluded.

### 2.3 Study design

**FIGURE 1:**
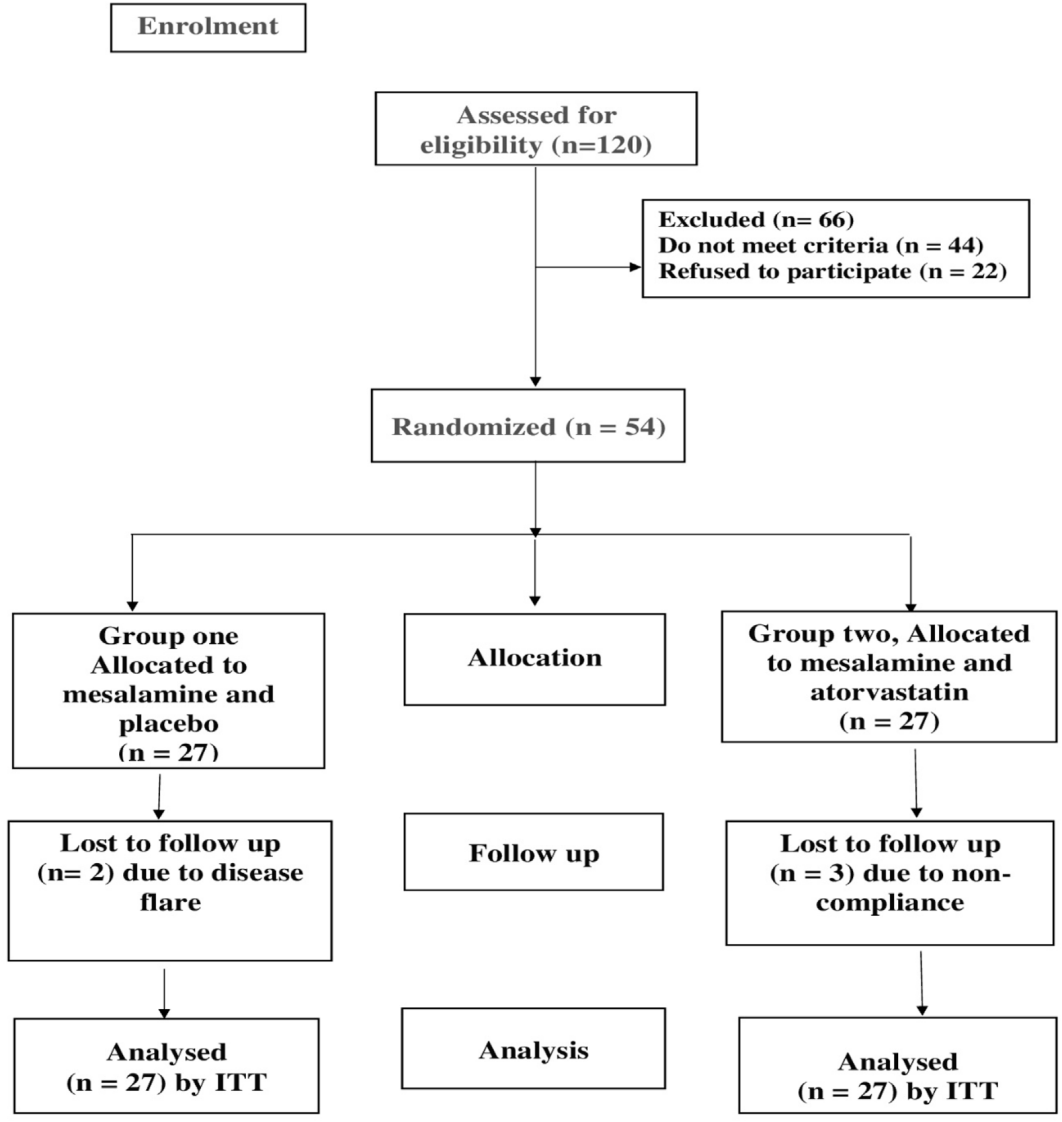
CONSORT diagram showing the flow of participants during the study (ITT, Intension to Treat).

In February 2023, this prospective, randomized, double-blind clinical trial was formally registered at ClinicalTrials.gov (identifier: NCT05567068). A total of 54 eligible participants were enrolled and randomly assigned to two study arms, as depicted in the CONSORT flow diagram (Figure 1). The allocation sequence was generated using a computer-based randomization method. Prior to participation, written informed consent was obtained from all patients.

Group 1 (control), participants were treated with 5-ASA tablets at a dose of 1 g three times daily, along with a once-daily placebo, for a total duration of 6 months (Pentasa^®^ 500 mg, Multi Pharm, Egypt).

Group 2 received the same 5-ASA regimen combined with atorvastatin tablets at a dose of 80 mg once daily for 6 months (Ator^®^ 80 mg, EPICO, Egypt). The placebo tablet was identical in appearance to the atorvastatin tablets and were manufactured by Zeta Pharma Company.

### 2.4 Sample size calculations

The primary hypothesis of this study was that adjunctive atorvastatin therapy would produce a greater reduction in the Simple Clinical Colitis Activity Index (SCCAI) and a greater improvement in Inflammatory Bowel Disease Questionnaire (IBDQ-32) scores compared with 5-ASA monotherapy. We identified prior clinical studies combining atorvastatin and 5-ASA (Alarfaj et al. 2025; AlRasheed et al. 2025b), but these used indices such as the Partial Mayo Score or Disease Activity Index, and did not report SCCAI effect sizes or standard deviations. Epidemiological studies and systematic reviews about the usage of statins in UC (Bai et al., 2021; Li et al., 2024; Lochhead et al., 2021) were also reported they are not directly suitable for estimating the effect size or standard deviation (SD) for SCCAI. Because SCCAI was our predefined primary clinical outcome, we were unable to derive reliable estimates for effect size or variance from existing literature. Consequently, this trial was designed as a pilot study, following the recommendations of Sim and Lewis (Sim and Lewis 2012) who suggested that a sample size of more than 22 participants per group is appropriate for detecting a small to medium effect size and reducing the combined error. Considering a potential dropout rate of 20%, the target sample size was increased to 27 patients per group. Participants were randomly assigned to each group, with the α-error set at 0.05 (two-tailed) and a statistical power of 0.80.

### 2.5 Study protocol

Patients were monitored closely throughout the 6-month study period to ensure treatment adherence and early detection of adverse events. Follow-up included monthly face-to-face clinic visits, during which clinical evaluation, assessment of disease activity, and laboratory investigations were performed. In addition, weekly follow-up phone calls were conducted to reinforce compliance, record any new symptoms, and address patient concerns. Hospital records were also reviewed regularly to capture any unreported adverse events or hospital admissions. Following the CONSORT recommendations, participants who met the inclusion criteria were allocated in a 1:1 ratio to either of the two treatment groups through a computer-generated randomization list. To maintain allocation concealment, the sequence was implemented using sequentially numbered, opaque, sealed envelopes. These envelopes were prepared by an independent research staff member who had no role in patient management, intervention delivery, or outcome assessment.

All medications were dispensed monthly in pre-labeled containers, and pill counts were performed at each visit to monitor adherence. The placebo tablets were formulated and packaged to be indistinguishable from the atorvastatin tablets in terms of size, shape, color, taste, and overall presentation to maintain blinding. Both patients and treating physicians were blinded to treatment allocation.

Participants in both groups were provided with standardized nutritional and lifestyle counseling at baseline, emphasizing balanced dietary habits and avoidance of major dietary changes during the study period. Patients were advised to refrain from using nonsteroidal anti-inflammatory drugs (NSAIDs), corticosteroids, immunosuppressive agents, or biologic therapies during the trial.

The selected 5-ASA dose (1 g t. i.d) was based on established treatment guidelines for mild-to-moderate UC (Sehgal et al., 2018) while the atorvastatin dose (80 mg once daily) was chosen based on prior clinical and preclinical studies demonstrating anti-inflammatory efficacy of atorvastatin (Aldossary et al., 2024).

The choice of atorvastatin at 80 mg once daily was based on evidence from cardiovascular and neurological trials where high-intensity statin regimens were required to achieve robust anti-inflammatory effects in addition to lipid lowering (Aldossary et al. 2024; Newman et al., 2006; Singh et al., 2008). This dose has demonstrated safety in various patient populations (Li et al., 2016) and is expected to achieve sufficient systemic exposure to modulate inflammatory pathways relevant to UC pathogenesis (AlRasheed et al. 2025b).

### 2.6 Study outcomes

#### 2.6.1 Primary outcomes

The study’s primary endpoint was the change in the simple clinical colitis activity index (SCCAI), a measure used to evaluate remission and clinical response in patients with mild to moderate UC.

#### 2.6.2 Secondary outcomes

Secondary endpoints encompassed variations in the Inflammatory Bowel Disease Questionnaire (IBDQ-32) scores, as well as alterations in serum IL-18 concentrations, erythrocyte sedimentation rate (ESR), and C-reactive protein (CRP) levels, together providing a comprehensive indication of the physiological and biological impact of atorvastatin therapy.

### 2.7 Follow-up

Participants were monitored through a structured follow-up schedule that included monthly face-to-face clinic appointments and weekly telephone check-ins. These interactions were used to evaluate treatment compliance, track symptom progression, and document any side effects or adverse reactions experienced during the study period. At the initial visit, a comprehensive baseline evaluation was performed, including a detailed medical history, complete physical examination, and laboratory investigations to exclude any underlying organic dysfunction. These investigations comprised a complete blood count, liver and kidney function tests. Inflammatory markers were also assessed, including serum levels of IL-18 and ESR, as well as CRP, which served as objective measures of intestinal inflammation. All laboratory tests were performed in the same certified laboratory to ensure consistency, and standardized ELISA kits were used for cytokine quantification following the manufacturer’s instructions.

### 2.8 Evaluation of colitis

The modified Simple Clinical Colitis Activity Index (SCCAI) is a validated tool used to assess disease activity in UC, relying solely on patient-reported symptoms without the need for endoscopic or laboratory parameters (Kishi et al., 2022). It comprises six clinical items: bowel frequency during the day (0–3), bowel frequency at night (0–2), urgency of defecation (0–3), presence of blood in stool (0–3), general wellbeing (0–4), and the presence of extra-intestinal manifestations (0–4). Each item is scored individually, and the total score ranges from 0 to 19, with higher scores indicating more severe disease activity. In general, a score of ≤4 reflects remission, 5–11 mild to moderate activity, and ≥12 severe activity. The SCCAI is widely used in both clinical practice and research because it is simple to administer, responsive to changes in disease status, and strongly correlates with physician global assessments. Its reliance on patient-reported outcomes also allows for repeated measurement over time without invasive procedures, making it particularly useful for monitoring treatment response in interventional studies.

A universally accepted numeric minimal clinically important difference (MCID) for the SCCAI has not been established. Based on prior validation and patient-vs-clinician studies of the SCCAI and its patient version (P-SCCAI), and consistent with thresholds used in previous clinical studies, we prespecified a decrease of ≥2 points from baseline in the SCCAI as the threshold for clinical response in this trial (Bennebroek Evertsz’ et al., 2013). This definition was used as the primary response criterion and is reported throughout the results.

### 2.9 Assessment of quality of life

The Inflammatory Bowel Disease Questionnaire-32 (IBDQ-32) is a disease-specific, patient-reported outcome measure designed to assess health-related quality of life in individuals with inflammatory bowel disease, including ulcerative colitis. It consists of 32 items grouped into four domains: digestive symptoms (10 items), systemic symptoms (5 items), emotional function (12 items), and social function (5 items). Each item is scored on a 7-point Likert scale, where 1 represents the worst function and 7 represents the best function over the preceding 2 weeks. The total score therefore ranges from 32 to 224, with higher scores indicating better quality of life. Domain scores can also be analyzed separately to capture specific aspects of disease impact. The IBDQ-32 is well-validated, sensitive to clinical changes, and correlates closely with objective measures of disease activity, making it a robust tool for evaluating treatment outcomes. Its comprehensive coverage of both physical and psychosocial dimensions allows for a holistic assessment of patient wellbeing in clinical trials and longitudinal studies (Alrubaiy et al., 2015; Chen et al., 2017).

### 2.10 Sample collection

Venous blood samples (10 mL) were obtained from the antecubital vein both at baseline and 6 months after initiating treatment. Each sample was transferred into collection tubes, allowed to clot at ambient temperature, and then centrifuged at 4,500 g for 10 min using a Hettich Zentrifugen EBA 20. The resulting serum was divided into two portions: one aliquot was stored at −80 °C for subsequent cytokine analysis, while the other was used immediately to assess routine liver and kidney function tests.

### 2.11 Biochemical analysis

Serum IL-18 (catalog no.: 201-12-0090) and CRP (catalog no. 514003) levels were measured using commercially available enzyme-linked immunosorbent assay (ELISA) kits (SunRed, Shanghai, China), following the manufacturer’s instructions. The CRP kit provided by Spectrum Diagnostics.

### 2.12 Statistical analysis

Data analysis was carried out using Prism software, version 9 (GraphPad Software, Inc., San Diego, CA, United States). The distribution of continuous variables was examined with the Shapiro–Wilk test to determine normality. For comparisons within each group, non-parametric variables were analyzed using the Wilcoxon signed-rank test, whereas parametric variables were assessed with the paired Student’s t-test, comparing pre- and post-treatment values. Differences between the two groups, both before and after the intervention, were analyzed using the Mann–Whitney U test for non-parametric data and the unpaired Student’s t-test for parametric data.

Categorical data were presented as frequencies and percentages, whereas continuous variables were reported as means ± standard deviation (SD) or medians with interquartile ranges (IQR), according to their distribution pattern. Associations between categorical variables were analyzed using the Chi-square test or Fisher’s exact test. For non-normally distributed data, correlations were assessed via Spearman’s rank correlation coefficient. All analyses were two-tailed, and statistical significance was defined as a p-value <0.05.

### 2.13 Multivariable and nonparametric multivariate methods

Because most continuous variables violated normality assumptions, we conducted the univariate nonparametric tests with multivariable and multivariate nonparametric analyses. For each continuous outcome (SCCAI total score, IBDQ domain scores, biomarker levels), we performed rank-based ANCOVA (i.e., the dependent variable and covariates were rank-transformed) with treatment as main effect, baseline rank, and covariates (age, sex, disease duration). For the set of IBDQ domain scores, we additionally applied a nonparametric multivariate test (rank-MANOVA) to assess global treatment effect, then conducted domain-specific rank-ANCOVAs if the omnibus test was significant. To control for multiplicity across multiple endpoints, p-values from domain-level tests were adjusted using the Benjamini–Hochberg false discovery rate (FDR) procedure. Adjusted effect estimates (on the rank scale) with 95% confidence intervals are reported. All tests were two-sided; α = 0.05 was used for the primary outcome, and FDR-adjusted p-values for secondary outcomes were used to interpret significance.

## 3 Results

### 3.1 Clinical, demographic and laboratory data of the patients

The baseline demographic, clinical, and laboratory characteristics of the two groups were comparable, with no statistically significant differences observed (Table 1). The mean age was 44.61 ± 12.35 years in the control group and 47.85 ± 8.65 years in the atorvastatin group (p = 0.269). The sex distribution (male/female) was similar between groups (14/13 vs. 12/15, p = 0.586). Mean body weight and BMI did not differ significantly (67.56 ± 4.91 kg vs. 68.19 ± 4.61 kg, p = 0.629; 22.35 ± 2.27 kg/m^2^ vs. 22.64 ± 2.11 kg/m^2^, p = 0.619). Liver function tests, including serum ALT and AST, showed no differences between the control and atorvastatin groups (ALT: 27.85 ± 6.17 IU/L vs. 29.04 ± 4.03 IU/L, p = 0.407; AST: 32.89 ± 6.74 IU/L vs. 32.85 ± 8.57 IU/L, p = 0.986). Renal function, assessed by serum creatinine, was also similar (0.919 ± 0.14 mg/dL vs. 0.953 ± 0.11 mg/dL, p = 0.334). Hemoglobin levels (12.23 ± 1.38 g/dL vs. 12.74 ± 1.24 g/dL, p = 0.153) and albumin concentrations (4.20 ± 1.14 g/dL vs. 4.27 ± 7.33 g/dL, p = 0.768) did not differ significantly. Median disease duration was comparable [0.9 years (0–2.2) vs. 1.2 years (0–2.9), p = 0.563], as was the proportion of treatment-naïve patients (8 vs. 7, p = 0.761) and smokers (9 vs. 7, p = 0.551). The distribution of disease extent was also similar between groups, with no significant differences in the proportions of patients with proctitis, left-sided colitis, or proctosigmoiditis (p = 0.534).

**TABLE 1:** Clinical, demographic and laboratory data of the patients.

Parameter	Control group (n = 27)	Atorvastatin group (n = 27)	P value
Age (years)	44.61 ± 12.35	47.85 ± 8.65	0.269
Sex (M/F)	14/13	12/15	0.586
Weight (kg)	67.56 ± 4.91	68.19 ± 4.61	0.629
BMI (kg/m^2^)	22.35 ± 2.27	22.64 ± 2.11	0.619
Serum ALT (IU/L)	27.85 ± 6.17	29.04 ± 4.03	0.407
Serum AST (IU/L)	32.89 ± 6.74	32.85 ± 8.57	0.986
SrCr (mg/dL)	0.919 ± 0.14	0.953 ± 0.11	0.334
Hgb (mg/dL)	12.23 ± 1.38	12.74 ± 1.24	0.153
Albumin (g/dL)	4.2 ± 1.14	4.27 ± 7.33	0.768
Disease duration	0.9 (0- 2.2)	1.2 (0- 2.9)	0.563
Naïve (no.)	8	7	0.761
Experience disease (no.)	19	20	
Smoking (no.)	9	7	0.551
Site of disease (no.)			
Proctitis	10	11	0.534
Left-sided	10	6	
Proctosigmoiditis	8	10	

Data was presented as mean ± SD, median, interquartile range, and numbers, Control group, UC patients treated with 5-ASA and placebo, Atorvastatin group, UC patients treated with 5-ASA plus atorvastatin, M, male; F, female; ALT, alanine aminotransferase; AST, aspartate aminotransferase; Hgb, haemoglobin, Sr Cr, serum creatinine. Significance at (*p* < 0.05).

### 3.2 Effect of study medications on simple colitis clinical activity index (SCCAI)

Over the course of follow-up, two participants from the control group discontinued due to disease exacerbation, while three from the atorvastatin group withdrew because of poor adherence. To maintain randomization integrity and minimize bias, the analysis followed the intention-to-treat (ITT) principle, applying the baseline observation carried forward (BOCF) approach to address missing data. All patients initially randomized were incorporated into the final statistical evaluation.

At baseline, SCCAI sub scores and total scores were comparable between groups. Within-group analysis, by Wilcoxon test, revealed significant improvements in most SCCAI parameters after treatment. In the placebo group, bowel frequency during the day decreased from a median of 2 (IQR 2–3) to 2 (IQR 1–2; p = 0.032), urgency of defecation from 2 (IQR 1–3) to 1.5 (IQR 1–2; p = 0.031), blood in stool from 2 (IQR 1–3) to 1 (IQR 1–2; p = 0.030), and general well-being from 2 (IQR 2–3) to 1 (IQR 1–2; p = 0.0005). No significant changes were observed in night-time bowel frequency or extraintestinal manifestations. The total SCCAI score in this group decreased significantly from 11 (IQR 9–11) to 7 (IQR 5–10; p < 0.0001).

In the atorvastatin group, significant improvements were observed across multiple parameters: bowel frequency during the day decreased from 2 (IQR 1–2) to 1 (IQR 0–2; p = 0.0017), night-time bowel frequency from 1 (IQR 0–1) to 0 (IQR 0–1; p = 0.001), urgency from 2 (IQR 1–3) to 1 (IQR 0–2; p = 0.003), blood in stool from 2 (IQR 1–3) to 1 (IQR 0–2; p = 0.008), and general wellbeing from 2 (IQR 1–2) to 1 (IQR 0–1; p = 0.004). Extraintestinal manifestations did not change significantly. The total SCCAI score declined from 9 (IQR 7–10) to 5 (IQR 4–6; p < 0.0005).

Between-group comparisons, by Man Whitney test, after treatment showed significantly greater improvement in the atorvastatin group compared to placebo in day-time bowel frequency (p = 0.024), night-time frequency (p = 0.023), urgency (p = 0.005), and total SCCAI score (p = 0.0005). Clinical response was achieved in 20 patients (66.6%) in the atorvastatin group versus 12 patients (44%) in the placebo group (p = 0.02). Remission rates were higher in the atorvastatin group (33.3% vs. 14.8%), though the difference did not reach statistical significance (p = 0.115) (Table 2).

**TABLE 2:** Effect of study medications on simple colitis clincal activity index.

Character	Control group (n = 27)			Atorvastatin group (n = 27)			*P* value
	Before treatment	After treatment	*P* value	Before treatment	After treatment	*P* value	After treatment
Bowel frequency (day)	2 (2-3)	2 (1-2)	0.032^*^	2 (1-2)	1 (0-2)	0.0017^*^	0.024^**^
Bowel frequency (night)	1 (0-2)	1 (0-2)	0.148	1 (0-1)	0 (0-1)	0.001^*^	0.023^**^
Urgency of defecation	2 (1-3)	1.5 (1-2)	0.031^*^	2 (1-3)	1 (0-2)	0.003^*^	0.005^**^
Blood in stool	2 (1-3)	1 (1-2)	0.03^*^	2 (1-3)	1 (0-2)	0.008	0.046
General wellbeing	2 (2-3)	1 (1-2)	0.0005^*^	2 (1-2)	1 (0-1)	0.004	0.027
Extra-intestinal manifestations	1 (0-2)	1 (0-1)	0.428	1 (0-2)	1 (0-1)	0.530	0.788
Total Score	11 (9-11)	7 (5-10)	<0.0001	9 (7-10)	5 (4-6)	<0.0001	0.0005
Response (n, %)	12 (44%)			20 (66.6%)			0.02^***^
Remission (n, %)	4 (14.8%)			9 (33.3%)			0.115

Data was presented as median, numbers, percentage, and interquartile range, Control group, UC, patients treated with 5-ASA, and placebo, Atorvastatin group; UC, patients treated with 5-ASA, plus atorvastatin. (^*^) level of significance within the same group using Wilcoxon test. (^**^) level of significance between groups using Mann Whitney test. (^***^) level of significance between groups using chi square test. Significance at (*p* < 0.05).

We conducted a *post hoc* power (sensitivity) analysis based on our observed SCCAI effect (rank biserial correlation r = −0.466), with total N = 54 and α = 0.05. The resulting estimated power is approximately 91%.

### 3.3 Effect of study medications on disease-specific quality of life (IBDQ subscales)

At baseline, IBDQ subscale scores were comparable between groups. Within-group analysis in the control group (5-ASA + placebo) showed significant improvements after treatment in the social domain (mean ± SD: 14.48 ± 5.85 to 18.81 ± 7.13; *p* = 0.002, paired *t*-test), systemic domain (15.04 ± 5.23 to 18.96 ± 3.65; *p* < 0.0001, Wilcoxon test), digestive domain [median (IQR): 32 (31–35) to 51 (36–58); *p* < 0.0001, Wilcoxon test], emotional domain [26 (18–36) to 34 (19–52); *p* = 0.007, paired *t*-test], and total IBDQ score (88.78 ± 16.94 to 118.5 ± 27.95; *p* < 0.0001, paired *t*-test).

In the atorvastatin group, significant post-treatment improvements were also observed in the social domain (15.7 ± 4.92 to 18.26 ± 5.87; *p* = 0.014, paired *t*-test), systemic domain (16.04 ± 4.84 to 22.3 ± 3.70; *p* < 0.0001, Wilcoxon test), digestive domain [33 (26–37) to 60 (47–65); *p* < 0.0001, Wilcoxon test], emotional domain [25 (14–42) to 52 (31–63); *p* = 0.001, paired *t*-test], and total IBDQ score (93.89 ± 19.89 to 141.9 ± 27.88; *p* < 0.0001, paired *t*-test).

Between-group comparisons after treatment showed that the atorvastatin group had significantly greater improvements in the systemic domain (*p* = 0.001, Mann–Whitney test), digestive domain (*p* = 0.013, Mann–Whitney test), emotional domain (*p* = 0.015, unpaired *t*-test), and total IBDQ score (*p* = 0.003, unpaired *t*-test), while the social domain improvement was not significantly different between groups (*p* = 0.755, unpaired *t*-test) (Table 3).

**TABLE 3:** Effect of study medications impact on disease-specific quality of life via IBDQ subscales.

Character	Control group (n = 27)			Atorvastatin group (n = 27)			*P* value
	Before treatment	After treatment	*P* value	Before treatment	After treatment	*P* value	After treatment
Social domain	14.48 ± 5.85	18.81 ± 7.13	0.002*	15.7 ± 4.92	18.26 ± 5.87	0.014*	0.755**
Systemic domain	15.04 ± 5.23	18.96 ± 3.65	<0.0001#	16.04 ± 4.84	22.3 ± 3.70	<0.0001#	0.001##
Digestive domain	32 (31-35)	51(36-58)	<0.0001#	33 (26-37)	60 (47-65)	<0.0001#	0.013##
Emotional domain	26 (18-36)	34 (19-52)	0.007*	25 (14-42)	52 (31-63)	0.001*	0.015**
Total IBDQ score	88.78 ± 16.94	118.5 ± 27.95	<0.0001*	93.89 ± 19.89	141.9 ± 27.88	<0.0001*	0.003**

Data was presented as median and interquartile range, Control group, UC, patients treated with 5-ASA, and placebo, Atorvastatin group; UC, patients treated with 5-ASA, plus atorvastatin, (^*^) level of significance within group using paired t-test. (^**^) level of significance between groups using unpaired t-test, (^#^) level of significance within group using Wilcoxon test. (^##^) level of significance between groups using Mann Whitney test, (IBDQ), inflammatory bowel disease questionnaire, Significance at (*p* < 0.05).

### 3.4 Effect of study medications on serum inflammatory parameters

At baseline, serum IL-18, CRP, and ESR levels were comparable between groups. Within-group analysis using the Wilcoxon test showed significant post-treatment reductions in all parameters for both groups. In the control group, IL-18 decreased from a median (IQR) of 189.7 (162.8–212.9) pg/mL to 159.7 (95–187.6) pg/mL (*p* = 0.005), CRP from 45.2 (27–71) mg/dL to 20.5 (15.8–47) mg/dL (*p* = 0.004), and ESR from 24 (19–26) mm/h to 13 (8–15) mm/h (*p* < 0.0001).

In the atorvastatin group, IL-18 levels fell from 183.4 (154.9–213.2) pg/mL to 86.12 (69.4–145.8) pg/mL (*p* < 0.0001), CRP from 41 (16–62.3) mg/dL to 16 (10–22.5) mg/dL (*p* = 0.0002), and ESR from 23 (19–26) mm/h to 8 (6–13) mm/h (*p* < 0.0001).

Between-group comparisons after treatment, analyzed using the Mann–Whitney test, demonstrated significantly lower IL-18 (*p* = 0.026), CRP (*p* = 0.027), and ESR (*p* = 0.03) levels in the atorvastatin group compared with the control group (Table 4).

**TABLE 4:** Effect of study medications on serum parameters.

Character	Control group (n = 27)			Atorvastatin group (n = 27)			*P* value
	Before treatment	After treatment	*P* value	Before treatment	After treatment	*P* value	After treatment
IL-18 (pg/mL)	189.7 (162.8-212.9)	159.7 (95-187.6)	0.005^a^	183.4 (154.9-213.2)	86.12 (69.4-145.8)	<0.0001^a^	0.026^b^
CRP (mg/dL)	45.2 (27-71)	20.5 (15.8-47)	0.004^a^	41 (16-62.3)	16 (10-22.5)	0.0002^a^	0.027^b^
ESR (mm/hr)	24 (19-26)	13 (8-15)	<0.0001^a^	23 (19-26)	8 (6-13)	0.0001^a^	0.03^b^

Data was presented as mean ± SD, control group; UC, patients treated with 5-ASA, and placebo, Atorvastatin group; UC, patients treated with 5-ASA, plus atorvastatin; IL-18, interleukin 18, CRP, c-reactive protein, ESR, erythrocyte sedimentation rate.

^a^ level of significance within thesame group by Wilcoxon test.

^b^ level of significance between groups using Man Witney test. Significance at (*p* < 0.05).

### 3.5 Adjusted treatment effects from rank-ANCOVA/multivariate analyses

In rank-based ANCOVA adjusted for baseline ranks, age, sex, and disease duration, treatment (atorvastatin vs. control) remained significantly associated with lower post-treatment SCCAI (β = −1.25, 95% CI –2.28 to −0.22, p = 0.018, pFDR = 0.025) as shown in Table 5. For inflammatory markers, rank-ANCOVAs showed significant adjusted effects for ESR (p = 0.031, pFDR = 0.042), IL-18 (p = 0.013, pFDR = 0.020), and CRP (p = 0.034, pFDR = 0.034) as shown in Table 5.

**TABLE 5:** Adjusted treatment effects from rank-ANCOVA/multivariate analyses.

Outcome	Adjusted estimate (β)	95% CI	p-value	pFDR (benjamini–hochberg)	Interpretation
SCCAI (post-treatment rank)	−1.25	−2.28 to −0.22	0.018	0.025	Atorvastatin group showed significantly lower (better) SCCAI after adjustment
ESR (post-treatment rank)	−1.05	−2.00 to −0.10	0.031	0.042	Significant adjusted reduction in ESR
IL-18 (post-treatment rank)	−1.47	−2.59 to −0.35	0.013	0.020	Significant adjusted reduction in IL-18
CRP (post-treatment rank)	−0.89	−1.71 to −0.07	0.034	0.034	Significant adjusted reduction in CRP

Simple clinical colitis activity index (SCCAI), erythrocyte sedimentation rate (ESR), C-reactive protein (CRP), and interleukin-18 (IL-18), CI, confidence intervals; FDR, false discovery rate.

### 3.6 Domain-level Rank-ANCOVA results for IBDQ-32

In our multivariable-adjusted analyses (rank-ANCOVA controlling for baseline domain rank, age, sex, and disease duration), the effect of atorvastatin remained significant for the digestive (β = −1.95, pFDR = 0.012) and systemic (β = −1.60, pFDR = 0.032) IBDQ domains, while the emotional domain exhibited a borderline adjusted improvement (β = −1.75, pFDR = 0.021). The social domain effect, though positive, did not meet significance after multiplicity correction (pFDR = 0.20) as shown in Table 6. The omnibus multivariate test (rank-MANOVA) confirmed a statistically significant overall treatment effect on the combined quality-of-life domains (Wilks’ λ, p = 0.004), supporting the notion that the group differences are not restricted to a single domain but span multiple, interrelated dimensions.

**TABLE 6:** Domain-level rank-ANCOVA results.

IBDQ-32 domain	Adjusted estimate (β)	95% CI	p (raw)	p (FDR-adjusted)	Significance
Digestive	−1.95	−3.20 to −0.70	0.004	0.012	Yes
Systemic	−1.60	−2.90 to −0.30	0.016	0.032	Yes
Emotional	−1.75	−3.10 to −0.40	0.007	0.021	Yes
Social	−0.85	−2.10 to 0.40	0.170	0.20	No

CI, confidence intervals; FDR, false discovery rate, inflammatory bowel disease questionnaire (IBDQ-32).

### 3.7 Correlation between measured variables in the atorvastatin group

**FIGURE 2:**
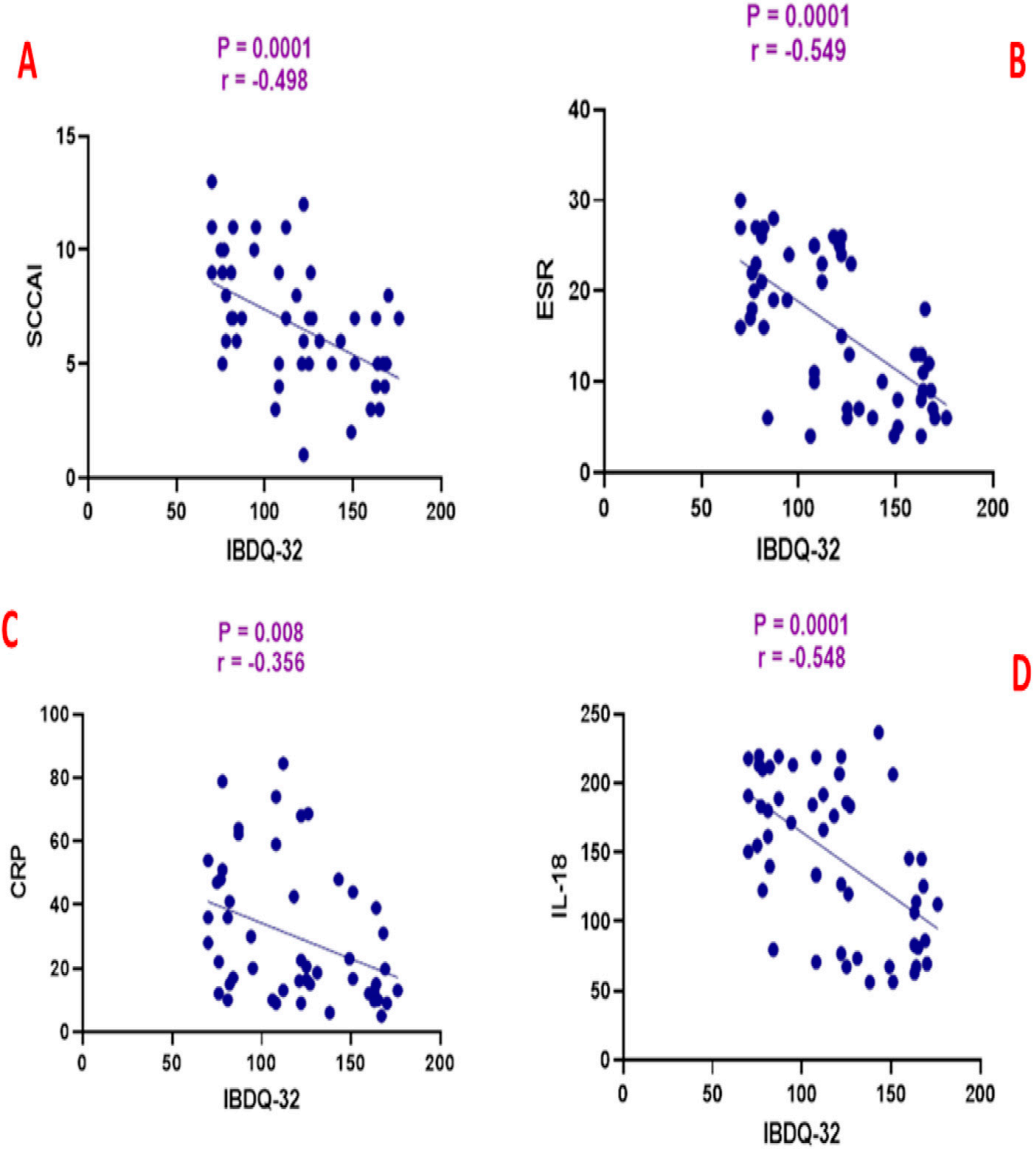
Correlation analysis between inflammatory bowel disease questionnaire (IBDQ-32) and **(A)** simple clinical colitis activity index (SCCAI), **(B)** erythrocyte sedimentation rate (ESR), **(C)** C-reactive protein (CRP), and **(D)** interleukin-18 (IL-18).

Spearman’s rank correlation analysis revealed significant associations between clinical, biochemical, and quality-of-life parameters in the atorvastatin group. Total IBDQ-32 scores showed significant negative correlations with SCCAI (*r* = −0.498, *p* = 0.0001), ESR (*r* = −0.549, *p* = 0.0001), CRP (*r* = −0.356, *p* = 0.008), and IL-18 (*r* = −0.548, *p* = 0.0001), indicating that higher quality-of-life scores were associated with lower disease activity and inflammatory markers as shown in Figure 2.

**FIGURE 3:**
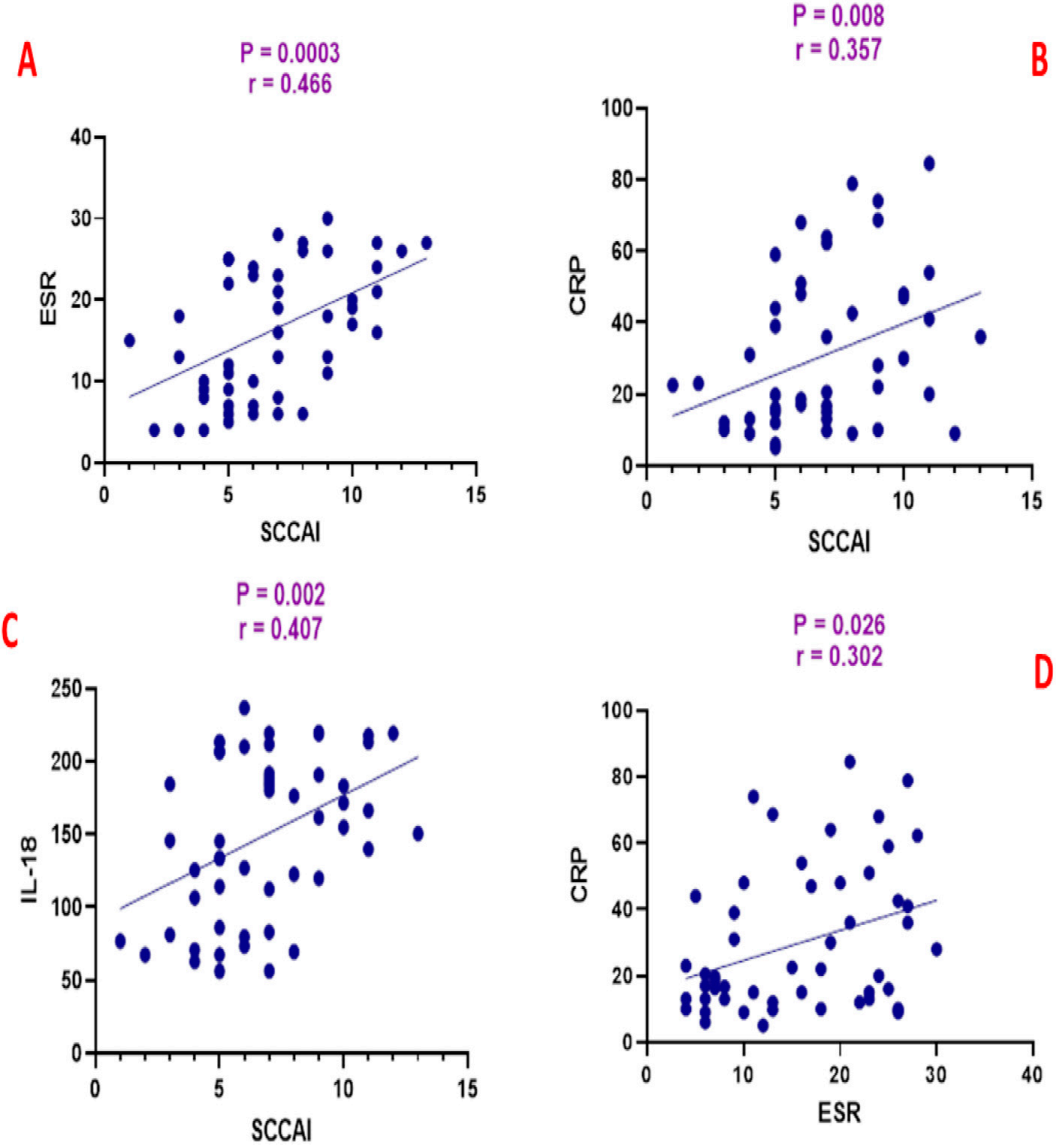
Correlation analysis between simple clinical colitis activity index (SCCAI) and **(A)** erythrocyte sedimentation rate (ESR), **(B)** C-reactive protein (CRP), and **(C)** interleukin-18 (IL-18), and **(D)** between ESR and CRP.

**FIGURE 4:**
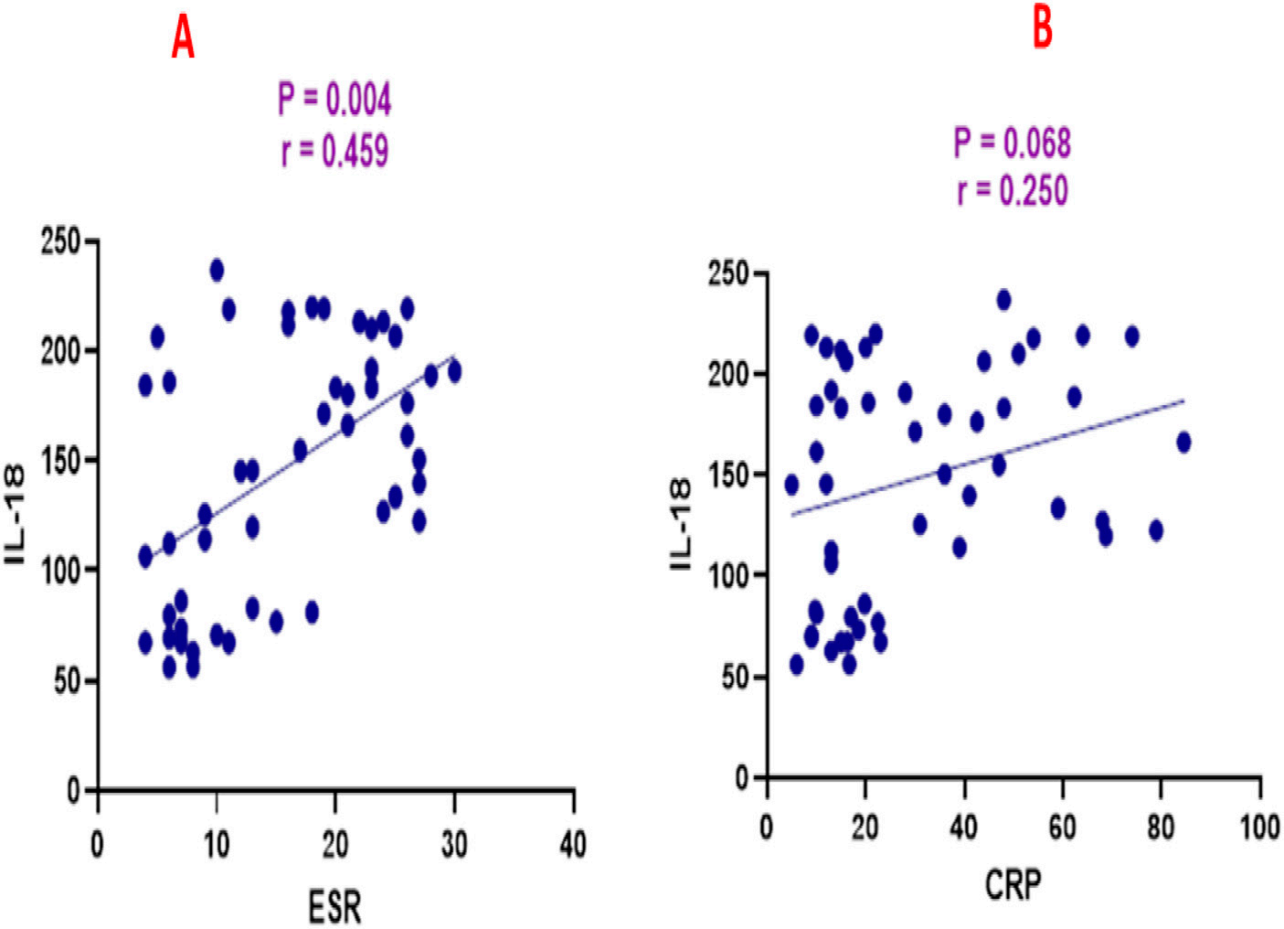
Correlation analysis between interleukin-18 (IL-18) and **(A)** erythrocyte sedimentation rate (ESR) and **(B)** C-reactive protein (CRP).

SCCAI scores were positively correlated with ESR (*r* = 0.466, *p* = 0.0003), CRP (*r* = 0.357, *p* = 0.008), and IL-18 (*r* = 0.407, *p* = 0.002) as shown in Figure 3. ESR was positively correlated with CRP (*r* = 0.302, *p* = 0.026) and IL-18 (*r* = 0.459, *p* = 0.0004) as shown in Figure 4. CRP and IL-18 showed a positive correlation trend (*r* = 0.250, *p* = 0.068), although this did not reach statistical significance.

### 3.8 Analysis of drug-related side effects

The incidence of drug-related side effects was generally low and comparable between groups, with no statistically significant differences detected using the chi-square test. In the atorvastatin group, reported adverse effects included vomiting in 4 patients (14.81%), nausea in 6 patients (22.22%), muscle weakness in 7 patients (25.9%), dizziness in 5 patients (18.51%), and headache in 5 patients (18.51%). In the control group, vomiting occurred in 3 patients (11.11%), nausea in 4 patients (14.81%), muscle weakness in 3 patients (11.11%), dizziness in 2 patients (7%), and headache in 4 patients (14.81%). None of these differences reached statistical significance (*p* > 0.05 for all comparisons) (Table 7).

**TABLE 7:** Analysis of drug related side effects between the studied groups.

Side effect	Control group; n = 27 (%)	Atorvastatin group; n = 27 (%)	*P* value
Vomiting	3 (11.11%)	4 (14.81%)	0.685
Nausea	4 (14.81%)	6 (22.22%)	0.483
Muscle weakness	3 (11.11%)	7 (25.9%)	0.161
Dizziness	2 (7%)	5 (18.51%)	0.224
Headache	4 (14.81%)	5 (18.51%)	0.715

Data was presented as numbers and percentages, Control group, UC, patients treated with 5-ASA, and placebo, Atorvastatin group; UC, patients treated with 5-ASA, plus atorvastatin, Significance at (p < 0.05) using Chi square test.

## 4 Discussion

Ulcerative colitis (UC) is characterized by persistent and recurrent inflammation of the gastrointestinal tract. Given its association with an elevated risk of colorectal cancer, there is an ongoing need to identify new therapeutic strategies that can slow disease progression, promote remission, and reduce relapse rates, ultimately enhancing patient outcomes (Abdin 2013).

Findings from a randomized clinical trial indicated that atorvastatin therapy showed no beneficial impact on acute UC flare-ups, and in certain cases, was associated with a paradoxical worsening of disease severity (Dhamija et al., 2014). In the referenced study, some participants exhibited higher disease activity following atorvastatin administration; however, the analysis did not include all eligible patients, and objective assessments—such as histopathology, endoscopic evaluation, and biochemical inflammatory markers—did not corroborate this observation. Evidence from multiple studies suggests that a higher atorvastatin dose (80 mg/day) is more effective than lower doses in reducing inflammation, with the high-dose group demonstrating a marked decline in inflammatory biomarkers (Singh et al. 2008; Subramanian et al., 2013). Moreover, Grip et al. reported that administering atorvastatin at 80 mg per day influenced both clinical manifestations and inflammatory markers in patients with Crohn’s disease (Grip et al., 2008). Thus, the limited efficacy of atorvastatin reported in the aforementioned study may be attributed to the use of a low dose (20 mg/day) and the brief intervention period of only 2 months. Such conditions may have been insufficient to elicit its anti-inflammatory effects or to mitigate disease exacerbation in UC. Recent studies have employed computational and epidemiologic approaches to explore repurposing statins in IBD: for example, Bai et al. (2021) used transcriptomic data and large EHR cohorts to identify atorvastatin as inversely correlated with UC gene signatures and demonstrated that atorvastatin use was associated with lower colectomy risk (Bai et al. 2021). Moreover, observational data such as Sun et al. (2023) have shown that statin use in IBD patients is associated with a reduced risk of colorectal cancer (Sun et al., 2023).

To our knowledge, this is the first clinical study to evaluate the effect of atorvastatin on SCCAI, IBDQ-32, and IL-18 in patients with mild to moderate UC. In the present study, adjunctive atorvastatin at 80 mg daily, when added to standard 5-ASA therapy, resulted in a clinically and statistically significant improvement in disease activity and disease-specific quality of life compared with placebo. The reduction in the modified SCCAI score was more pronounced in the atorvastatin group, while the placebo group showed only partial improvement. This greater reduction suggests that atorvastatin’s benefits extend beyond symptomatic relief, potentially influencing underlying inflammatory pathways. These results were matched with previous reports (AlRasheed et al. 2025b; Sonkar and Sachdeva 2022). The observed improvement may be attributed to the additional anti-inflammatory actions of 5-ASA and atorvastatin, which help relieve gastrointestinal symptoms and support mucosal recovery. These results are consistent with earlier studies suggesting a therapeutic role for statins in IBD (El-Mahdy et al. 2021; Maheshwari et al., 2015; Sasaki et al., 2003a). Our findings indicate that atorvastatin could be beneficial for patients with mild-to-moderate UC by alleviating clinical symptoms. In experimental models of colitis, atorvastatin treatment markedly lowered colonic endoscopic scores and enhanced both histological and immunohistochemical outcomes relative to baseline. These observations point toward a possible role for atorvastatin in regulating intestinal inflammation and facilitating mucosal repair in UC (El-Mahdy et al. 2021). EL-Mahdy et al. demonstrated that combining atorvastatin with 5-ASA significantly reduced the disease activity index (DAI) in an oxazolone-induced colitis model (El-Mahdy et al. 2021). In the work of El-Mahdy et al., treatment of experimental colitis with atorvastatin was associated with increased levels of the anti-inflammatory cytokine IL-10, supporting its anti-inflammatory potential. The investigators also observed a significant enhancement and upregulation of tight junction proteins following atorvastatin administration (El-Mahdy et al. 2021). These results collectively demonstrate the protective role of atorvastatin in reducing disease activity index scores and mitigating inflammation in patients with UC.

The marked improvement in IBDQ-32 total and subdomain scores further reinforces atorvastatin’s impact. The magnitude of change observed in the atorvastatin group exceeds the placebo group, indicating a change that is both statistically significant and meaningful from the patient’s perspective. Enhanced scores in emotional domains highlight that the benefits are not limited to gastrointestinal symptom relief, but also encompass psychological well-being and social functioning — domains often neglected in conventional therapeutic evaluation. The beneficial effects on IBDQ-32 domains can be similarly explained. By attenuating systemic and local inflammation, atorvastatin may alleviate fatigue, abdominal discomfort, and other systemic manifestations of UC, enabling patients to engage more fully in social and occupational activities. Moreover, improved emotional and social well-being in chronic disease has been linked not only to symptom control but also to decreased systemic inflammatory burden, suggesting that biochemical and psychosocial improvements may be interrelated.

Comparative clinical studies have reported similar trends. For example, observational cohorts have found that statin use in IBD correlates with reduced disease flare rates, fewer hospitalizations, and better patient-reported outcomes (Crockett et al. 2012; Khalili et al. 2025). Although data from randomized controlled trials remain limited, smaller pilot studies have indicated that statins, when added to standard therapy, may enhance remission rates and quality-of-life measures (Alarfaj et al. 2025; AlRasheed et al. 2025b). Importantly, the observed clinical benefits in UC parallel findings from other chronic inflammatory conditions, such as rheumatoid arthritis and multiple sclerosis, where statins have shown both symptom reduction and functional improvement (Kamm et al., 2012; McCarey et al., 2004).

Inflammatory biomarkers provide an objective correlate to the clinical outcomes in UC, and changes in their levels can shed light on the underlying mechanisms of therapeutic interventions. Our study indicated that addition of atorvastatin to 5-ASA significantly reduced serum IL-18, ESR, and CRP. These results were in line with previous reports (Alarfaj et al. 2025; Coward et al., 2006; Elkatary et al., 2015). IL-18 is a pro-inflammatory cytokine of the IL-1 family, produced predominantly by activated macrophages, epithelial cells, and dendritic cells in the intestinal mucosa (Coward et al. 2006; Sivakumar et al., 2002). It plays a pivotal role in UC pathogenesis by promoting Th1 and Th17 immune responses, stimulating IFN-γ production, and enhancing neutrophil recruitment to inflamed tissue. Elevated IL-18 levels have been documented in serum, colonic tissue, and stool of patients with active UC, correlating with disease severity (Sivakumar et al. 2002; Williams et al., 2019). Experimental studies suggest that atorvastatin can downregulate IL-18 production through inhibition of NLRP3 inflammasome activation, suppression of caspase-1–mediated IL-18 maturation, and blockade of downstream NF-κB signaling (Sun et al., 2020). These molecular effects have been observed in models of colitis and other inflammatory diseases, aligning with the hypothesis that statin therapy could attenuate mucosal cytokine overexpression in UC (Aktunc et al., 2011).

C-reactive protein (CRP), an acute-phase reactant synthesized by the liver in response to IL-6 and other pro-inflammatory cytokines, is a widely used systemic marker of inflammation in IBD (Takač et al., 2020). In UC, CRP levels are generally lower than in Crohn’s disease but still rise during moderate-to-severe activity and correlate with endoscopic inflammation in many patients (Miranda-García et al., 2016). Statins have been shown to reduce CRP concentrations in both cardiovascular and non-cardiovascular populations, independent of their lipid-lowering action (Morofuji et al., 2022). Mechanistically, this effect is attributed to inhibition of hepatic CRP synthesis through upstream cytokine suppression and modulation of STAT3 signaling (Kandelouei et al., 2022). The reduction in CRP with atorvastatin may therefore reflect a systemic anti-inflammatory effect that complements mucosal healing (Grip et al. 2008).

Erythrocyte sedimentation rate (ESR) reflects the presence of circulating acute-phase proteins such as fibrinogen, which increase red blood cell aggregation (Simeon et al., 2024). In UC, elevated ESR is associated with active inflammation, anemia, and chronicity of disease (Turner et al., 2011). Although ESR is less responsive to rapid changes in inflammation compared to CRP, it remains useful for monitoring long-term inflammatory trends. Statin therapy may lower ESR indirectly by decreasing hepatic synthesis of fibrinogen and other acute-phase proteins through anti-cytokine effects (Sarabi et al., 2016). This has been observed in patients with chronic inflammatory conditions such as rheumatoid arthritis, where atorvastatin treatment was associated with ESR reduction alongside clinical improvement (Sarabi et al. 2016).

The adjusted rank-ANCOVA results affirm the therapeutic benefit of adding atorvastatin to 5-ASA: after controlling for baseline values, age, sex, and disease duration, the treatment group exhibited significantly lower post-treatment SCCAI. Among inflammatory biomarkers, atorvastatin produced significant rank-adjusted reductions in ESR, IL-18, and CRP. These aligned biochemical changes support the clinical improvement inferred from SCCAI. In quality-of-life domains, the multivariate rank-MANOVA showed a significant global effect, and domain-specific rank-ANCOVAs confirmed significant adjusted benefits in the digestive emotional and systemic domains. Taken together, these findings suggest that the atorvastatin + 5-ASA regimen exerts a coherent effect across multiple axes: symptomatic disease activity, systemic inflammation, and patient-reported wellbeing. The consistency between clinical, biomarker, and quality-of-life outcomes strengthens confidence that the observed effects are not simply due to chance or confounding.

However, some caution is warranted. Because analyses were conducted on rank-transformed outcomes, effect estimates reflect relative ordering rather than raw score changes. The social domain’s failure to remain significant after FDR correction may indicate weaker or more variable responsiveness. Moreover, even though we adjusted for several covariates, residual confounding cannot be excluded. Finally, the lack of endoscopic data limits our ability to directly link these changes to mucosal healing. Nonetheless, the integration of multiple adjusted analyses affords a more robust and nuanced interpretation, and these findings support the rationale for future larger trials to confirm and expand upon the effects of atorvastatin adjunct therapy in UC.

Biologic agents (such as infliximab, adalimumab, vedolizumab, and others) have demonstrated high efficacy in inducing remission, mucosal healing, and sustaining response in moderate-to-severe UC (Bonovas et al., 2018). Meta-analyses and network trials consistently show that biologics outperform placebo and some traditional therapies in such populations (Singh et al., 2018). However, their cost and resource requirements (infusions, monitoring, and risk of immunogenicity) are substantial (Papamichael et al., 2023). In contrast, atorvastatin is inexpensive, orally administered, and widely accessible. Our findings—that atorvastatin added to 5-ASA produces statistically and clinically meaningful improvements in SCCAI, IBDQ domains, and inflammatory markers—suggest that atorvastatin could offer a low-cost adjunctive strategy, particularly in resource-limited settings or for patients for whom biologics are not feasible. Further larger studies comparing atorvastatin directly or in combination with biologic regimens would be valuable.

Taken together, the modulation of IL-18, CRP, and ESR by atorvastatin may represent a multifaceted anti-inflammatory action — targeting both upstream cytokine activation and downstream acute-phase responses. These changes not only provide biological plausibility for the observed clinical benefits but also support the concept that statins may address both local mucosal and systemic inflammation in UC.

Given that baseline demographic and clinical characteristics were comparable between groups, the observed therapeutic effects can be attributed mainly to the administered treatments. In this study, patients in the placebo arm exhibited improvements in SCCAI scores and significant reductions in serum ESR, CRP, and IL-18 relative to baseline. Furthermore, their IBDQ-32 scores increased notably compared with initial measurements. These benefits are most likely due to 5-ASA, a standard therapy for mild-to-moderate UC (Karagozian and Burakoff 2007). These findings are consistent with earlier research examining the impact of 5-ASA in experimental colitis models (El-Mahdy et al. 2021; Rashidian et al. 2016).

Our study builds upon and complements previous clinical investigations evaluating the therapeutic potential of atorvastatin combined with 5-ASA in UC, such as those by Alarfaj et al. (2025) and AlRasheed et al. (2025a), which demonstrated improvements in inflammatory and clinical outcomes (Alarfaj et al. 2025; AlRasheed et al. 2025b). Distinct from these earlier trials, the present study represents one of the few randomized, placebo-controlled investigations exploring the adjunctive use of high-dose atorvastatin (80 mg daily) in UC. We adopted a comprehensive evaluation strategy encompassing both clinical indices (SCCAI, IBDQ-32) and biochemical markers (IL-18, CRP, ESR), thereby integrating patient-reported outcomes with objective measures of inflammation.

Our methodological approach further advances prior work through the inclusion of an expanded biomarker panel—particularly IL-18—as well as rigorous statistical analyses, including rank-ANCOVA, sensitivity testing, and false discovery rate (FDR) correction, to address potential imbalances and control for multiple comparisons. The application of both parametric and non-parametric methods ensured appropriate handling of data based on distributional characteristics, enhancing analytical robustness. By employing a relatively high statin dose, this study also explores the potential ceiling of atorvastatin’s anti-inflammatory effects, providing new insights into dose-response relationships in inflammatory bowel disease.

While acknowledging that the atorvastatin–5-ASA combination has been previously examined, the novelty of our work lies in its methodological rigor, analytical depth, and mechanistic scope. These refinements strengthen the evidence supporting the combination’s therapeutic promise and underscore the need for future large-scale studies directly comparing atorvastatin alone, 5-ASA alone, and their combination to delineate potential additive interactions.

Our study has certain limitations that must be acknowledged. The trial was conducted at a single center with a relatively small sample size, which may limit the generalizability of the findings to broader UC populations. The follow-up period was relatively short, precluding assessment of long-term efficacy, sustainability of benefits, and impact on relapse rates. The study design did not include endoscopic or histologic evaluation, which are considered gold-standard measures of mucosal healing in UC. Furthermore, while atorvastatin’s pleiotropic effects are well-recognized, the mechanistic pathways underlying the observed clinical and biochemical changes were not directly investigated through molecular assays. Potential confounding factors such as diet, adherence variability, and concurrent lifestyle modifications were not systematically controlled.

The lack of standardized prior estimates for SCCAI in earlier studies limited our ability to perform a more precise sample size calculation; our trial was therefore a pilot designed to generate those estimates. Due to the absence of an *a priori* sample size calculation based on SCCAI, we have included a *post hoc* power (sensitivity) analysis using the observed rank biserial correlation for SCCAI. This estimated power suggests that our study had reasonable ability to detect effects of that magnitude. However, because this calculation is retrospective, based on nonparametric measures and parametric approximations, it should be interpreted with caution.

## 5 Conclusion

Adjunctive therapy with high-dose atorvastatin (80 mg once daily) in patients with UC receiving standard 5-ASA treatment was associated with marked improvements in clinical disease activity, as assessed by the modified SCCAI, and in disease-specific quality of life measured by the IBDQ-32. These clinical benefits were accompanied by significant reductions in key systemic and mucosal inflammatory markers, including IL-18, CRP, and ESR, supporting the hypothesis that atorvastatin exerts anti-inflammatory effects beyond its lipid-lowering properties.

While the findings are encouraging, they should be interpreted in light of the study’s modest sample size, single-center design, and short follow-up duration. Larger, multicenter trials with extended observation periods, incorporation of endoscopic and histological endpoints, and exploration of long-term safety are required to validate these results and refine dosing strategy. We recommend clinical trials with atorvastatin only treatment group to investigate the synergistic effect between 5-ASA and atorvastatin.

## Data Availability

The raw data supporting the conclusions of this article will be made available by the authors, without undue reservation.
